# A comparison of liver protection among 3-D conformal radiotherapy, intensity-modulated radiotherapy and RapidArc for hepatocellular carcinoma

**DOI:** 10.1186/1748-717X-9-48

**Published:** 2014-02-06

**Authors:** Dong Chen, Renben Wang, Xiangjiao Meng, Tonghai Liu, Hongjiang Yan, Rui Feng, Shangang Liu, Shumei Jiang, Xiaoqing Xu, Kunli Zhu, Xue Dou

**Affiliations:** 1 Department of Radiation Oncology, Shandong Cancer Hospital, Jinan, Shandong 250117, China; 2 School of Medicine and Life Sciences, University of Jinan-Shandong Academy of Medical Sciences, Shandong Cancer Hospital, Jinan, Shandong 250117, China

**Keywords:** Hepatocellular carcinoma, Radiotherapy, Dosimetry, Radiation-induced liver disease, Liver protection

## Abstract

**Purpose:**

The analysis was designed to compare dosimetric parameters among 3-D conformal radiotherapy (3DCRT), intensity-modulated radiotherapy (IMRT) and RapidArc (RA) to identify which can achieve the lowest risk of radiation-induced liver disease (RILD) for hepatocellular carcinoma (HCC).

**Methods:**

Twenty patients with HCC were enrolled in this study. Dosimetric values for 3DCRT, IMRT, and RA were calculated for total dose of 50 Gy/25f. The percentage of the normal liver volume receiving >40, >30, >20, >10, and >5 Gy (V_40_, V_30_, V_20_, V_10_ and V_5_) were evaluated to determine liver toxicity. V_5_, V_10_, V_20_, V_30_ and D_mean_ of liver were compared as predicting parameters for RILD. Other parameters included the conformal index (CI), homogeneity index (HI), and hot spot (V_110%_) for the planned target volume (PTV) as well as the monitor units (MUs) for plan efficiency, the mean dose (D_mean_) for the organs at risk (OARs) and the maximal dose at 1% volume (D_1%_) for the spinal cord.

**Results:**

The D_mean_ of IMRT was higher than 3DCRT (p = 0.045). For V_5_, there was a significant difference: RA > IMRT >3DCRT (p <0.05). 3DCRT had a lower V_10_ and higher V_20_, V_30_ values for liver than RA (p <0.05). RA and IMRT achieved significantly better CI and lower V_110%_ values than 3DCRT (p <0.05). RA had better HI, lower MUs and shorter delivery time than 3DCRT or IMRT (p <0.05).

**Conclusion:**

For right lobe tumors, RapidArc may have the lowest risk of RILD with the lowest V_20_ and V_30_ compared with 3DCRT or IMRT. For diameters of tumors >8 cm in our study, the value of D_mean_ for 3DCRT was lower than IMRT or RapidArc. This may indicate that 3DCRT is more suitable for larger tumors.

## Introduction

Hepatocellular carcinoma (HCC) is the third cause of cancer related death following lung and stomach cancer [1]. Resection and liver transplantation are generally regarded as curative treatments for HCC in the early stage and have shown effective results [2]. However, surgical resection accompanies high recurrence rate, and transplantation cannot be universally applicable. Now Radiotherapy technology has evolved remarkably and plays an important role in the treatment of HCC. During the past decade, improvement of survival had been observed from a high increase of radiation dose [3,4]. However, a high radiation dose to the liver would give rise to acute and late hepatic toxicity. Radiation-induced liver disease (RILD) is the most severe radiation-induced complication which may result in hepatic failure and death. The occurrence of RILD is associated with Child-Pugh grade, hepatic cirrhosis and the volume of liver receiving radiotherapy (RT). Cheng et al. [5] showed that both Child-Pugh Class B and the presence of hepatitis B virus were associated with the risk of RILD. What is more, chronic infection with HBV is responsible for 60% of HCC in Asia and Africa [6]. In Liang et al.’s study [7], the severity of hepatic cirrhosis was proved to be a unique independent predictor for RILD. Son et al. [8] suggested that the total liver volume receiving <18Gy should be greater than 800 cm^3^ to reduce the risk of the deterioration of hepatic function. Therefore, the study of predicting parameters for RILD risks and sparing more normal liver during RT is essential for HCC patients.

Now 3DCRT can irradiate the target volume accurately while minimizing the dose to normal liver and may offer a chance of long survival for some HCC patients [9]. With the development of an advanced form of 3DCRT, intensity-modulated radiotherapy (IMRT) can improve radiation plan quality by using an inverse planning algorithm to generate complex spatial dose distributions to conform more closely to the target volume. Recent years, RapidArc (RA) was developed to improve the time efficiency of dose delivery and produce highly conformal dose spacial distribution by changing treatment apertures (defined by dynamic multiple leaf collimators) and a modulated dose rate [10]. Poon et al. [11] have reported a significant improvement in sparing OAR and better conformity using RA compared with IMRT. But others may not. Kan et al. [12] showed that double-arc RA plans produced slightly inferior parotid sparing and dose homogeneity than IMRT. The purpose of this study was to compare the predicting parameters for RILD among 3DCRT, IMRT and RA for HCC.

## Methods

### Patient selection

Patients who underwent RT for primary HCC were registered and the database was retrospectively reviewed from January 2010 to March 2013 at Shandong Cancer Hospital. Eligibility criteria were as follows: (1) All patients underwent alpha-fetoprotein examination, contrast-enhanced computed to tomography, and ultrasonography to confirm the diagnosis. (2) No one had cirrhosis or portal vein thrombosis; (3) All patients had centrally located lesions on the right liver lobe; (4) Computed tomography scanning included whole liver, and bilateral kidney with a 3-mm slice thickness. (5) The patients experienced transarterial chemoembolization (TACE) or not. Informed consent was obtained from all patients, and the local Ethical Board approved the study protocol (Shandong tumor prevention and control institute ethics committee).

### Target delineation and planning techniques

The patients were fixed using vacuum casts in a supine position with both arms raised above their heads. There was no respiratory control training or other means to decrease degree of excursion of the liver. We defined the gross tumor volume (GTV) as the volume of primary tumor evident on contrast-enhanced CT images. The clinical target volume (CTV) was delineated on the basis of the GTV expanded by 5 mm. The planning target volume (PTV) was defined as the CTV with a 5-mm radial expansion and a 10-mm craniocaudal expansion to account for errors caused by the daily setup process and internal organ motion [13]. The OARs considered were healthy liver (whole liver minus PTV), kidneys, spinal cord and stomach. The target delineation was performed by the same experienced oncologist. Three sets of plans were all designed on the Varian Eclipse version 8.6.23 treatment planning system which was equipped with a Millennium multileaf collimator (MLC) (Varian) with 120 leaves. For 3DCRT and IMRT plans, all the gantry angles and radiation fields were confirmed according to the relationship of the PTVs and OARs to different situations, and the number of fields varied from 4 to 7. For RA, the plan was generated using two arcs rotating from 55° to 181° anticlockwise and from 181° to 55° clockwise with the dose rate varied between 0 MU/min and 600 MU/min (upper limit). A fixed DR of 300 MU/min was selected for IMRT and 3DCRT. All three sets of plans were designed by the same experienced physicist using 6- or 15-MV photon beams.

### Planning objectives and evaluation tools

The total prescribe dose was 50 Gy/25f. The planning objectives were to cover at least 95% of the PTV with the 90% isodose, to have minimum dose > 90% and maximum dose <110%. All plans were normalized to the mean dose of PTV to avoid any bias. For OARs, the tolerated maximum dose of spinal cord was 40 Gy. The mean dose of liver was limited to 30 Gy and V_30_ <50%. The mean dose of kidneys were 23 Gy (at least one side) and V20 <20%, the mean dose of stomach <20 Gy [13,14]. For PTV, V_x%_ means the volume receiving ≥ x% of the prescribed dose. For example, the V_95%_ means the volume receiving at least 95% of the prescribed dose and V_110%_ is used to represent the hot spot in the PTV. The conformal index (CI) = V_t,ref_/V_t_ × V_t,ref_/V_ref_,where V_t_ was the volume of PTV, V_ref_ was the volume enclosed by the prescription dose line, and V_t,ref_ is the volume of PTV within V_ref_[15]. The target homogeneity was defined as: HI = D_5%_/D_95%_ where D_5%_ and D_95%_ are the minimum doses delivered to 5% and 95% of the PTV [16,17]. The value of HI and CI range from 0 to 1. The more approximate to 1, the better [18].

For OARs, the parameters included the mean dose, the maximum dose expressed as D_1%_ and a set of appropriate V_x_, and D_y_, where V_x_ means the volume of the OARs receiving the dose > x Gy. For example, V_5_ of liver means the volume of normal liver receiving >5 Gy and presents low-dose exposure for the normal liver. D_1%_ of spinal cord presents the maximum dose spinal cord received.

What is more, the number of monitor units (MUs) per fraction and beam-on time were also analyzed to compare the efficiency of three sets of the plans. The treatment delivery time was defined as the time recorded between beam-on for the first field and beam-off for the last field.

### Statistics analysis

The statistical significance of difference in the outcome between the three techniques was evaluated using Paired *t*-test. All statistical tests were two-tailed and the software performed for assessment was SPSS 13.0 for Windows (SPSS Inc, Chicago, Illinois, USA). P < 0.05 was considered significant.

## Results

### Patient characteristics

**Figure 1:**
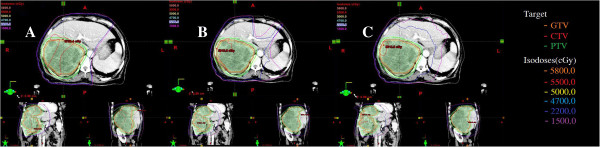
**Isodose curves on axial, coronal, and sagittal views for one representative case of larger tumor. A**: 3DCRT, **B**: IMRT and **C**: RA. RA achieved better conformality compared with 3DCRT and IMRT.

**Figure 2:**
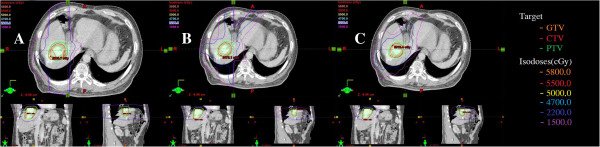
**Isodose curves on axial, coronal, and sagittal views for one representative case of smaller tumor. A**: 3DCRT, **B**: IMRT and **C**: RA. RA achieved better conformality compared with 3DCRT and IMRT.

**Figure 3:**
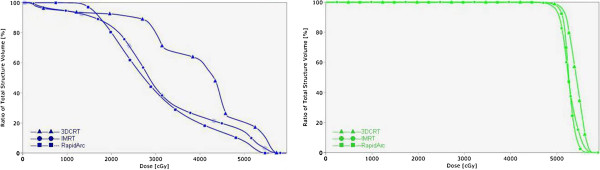
**The comparison of DVHs for normal liver in 3DCRT, IMRT and RA for the larger tumor.** Right figure: DVHs of PTV. These three techniques produced similar homogeneity of the PTV and 3DCRT obtained highest volume of hot spot. Left figure: DVHs of normal liver. RA obtained the highest low-dose distribution in the normal liver compared with 3DCRT and IMRT. However, 3DCRT obtained the highest high-dose distribution in the normal liver compared with IMRT and RA.

**Figure 4:**
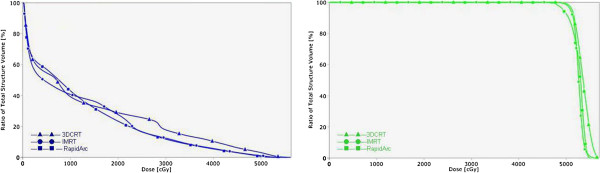
**The comparison of DVHs for normal liver in 3DCRT, IMRT and RA for the smaller tumor.** Right figure: DVHs of PTV. These three techniques produced similar homogeneity of the PTV and 3DCRT obtained highest volume of hot spot. Left figure: DVHs of normal liver. These three techniques produced similar low-dose distributions the liver. 3DCRT obtained the highest V_20_ and V_30_, whereas no statistical difference was observed.

The characteristics of patients are summarized in Table 1. There were 16 males and 4 females, and their median age was 60 years (range, 41–65 years). The PTV was 775.39 ± 361.98 (range, 107.53-3568.03 cm^3^). We divided our patients into two groups according to the median value (D = 8 cm) of the tumor diameter. There was no whole liver included into the PTVs. Table 2 showed the results with the mean value ± standard deviation for the considered parameters of OARs. Table 3 showed the parameters of dose-volume histograms (DVHs) with the mean value ± standard deviation for PTV, MU and delivery time. Table 4 showed the predictive parameters for RILD with the mean value ± standard deviation of three techniques for larger (D > 8 cm) and smaller (D ≤ 8 cm) tumors of our study. Figures 1 and 2 showed the dose distributions of two examples for axial, sagittal, and coronal views for smaller and larger tumors. Figures 3 and 4 showed DVHs of the PTVs and healthy liver compared among the three plans for the patients corresponding with Figures 1 and 2.

**Table 1:** Patient characteristics and tumor parameters

**Variables**	**No. of patients/volume**
Gender	
Male	16
Female	4
Age (years)	
Median	60
Range	41–65
Viral etiology	
HBs-Ag (+)	15
HBs-Ag (-)	5
Child-Pugh class	
A	14
B	6
GTV (cm^3^)	
Median (Range)	753.11 (34.54–2125.72)
Mean ± SD	526.89 ± 226.24
Equiv. Sphere Diameter (cm)	
Median (Range)	8.0 (4.3–17.0)
Mean ± SD	7.5 ± 1.73
PTV (cm^3^)	
Median	533.87 (107.53–3568.03)
Mean ± SD	775.39 ± 361.98

*Abbreviations*: *HBs-Ag* hepatitis B surface-antigen, *SD* standard deviation, *GTV* gross tumor volume, *PTV* planning target volume.

**Table 2:** Summary of the dosimetric results for OARs

	**3DCRT**	**IMRT**	**RA**	**P-value**		
				**a**	**b**	**c**
Liver D_mean_ (Gy)	20.57 ± 7.12	22. 34 ± 7.33	20.51 ± 7.12	0.045	0. 051	0.060
Liver V_5_ (%)	68.9 ± 19.23	70.43 ± 18.92	76.34 ± 19.12	0.02	0.015	0.007
Liver V_10_ (%)	60.37 ± 21.54	65.12 ± 21.62	64.71 ± 21.63	0.274	0.031	0.004
Liver V_20_ (%)	48. 34 ± 21.13	47.73 ±22.81	43. 94 ± 20.10	0.34	0.23	0.012
Liver V_30_ (%)	22.27 ±17..30	22.57 ± 15.73	21.93 ±14..30	0.002	0.450	0.013
Liver V_40_ (%)	27.73 ± 18.73	17.94 ±10.13	17.93 ± 10.24	0.012	0.453	0.038
Stomach D_mean_ (Gy)	14.3 ± 13.93	14.36 ±10.13	16.13 ±12..34	0.231	0.937	0.073
Left kidney D_mean_ (Gy)	2.03 ± 2.45	2.13 ±2.98	2.01 ±2.94	0.45	0.270	0.110
Right kidney D_mean_ (Gy)	6.73 ±8.96	5.13 ± 6.73	4.36 ±6.58	0.134	0.078	0.734
Spinal cord D_1%_ (Gy)	20.20 ± 8.34	19.23 ± 9.70	14.23 ± 7.92	0.721	0.210	0.372

Statistical significance (p <0.05) was reported between couples from paired *t*-test analysis. *Abbreviations: 3DCRT* 3-D conformal radiation therapy, *IMRT* intensity-modulated radiation therapy, *RA* RapidArc. *V*_*x*_ the volume of the OARs receiving the dose > x Gy. *D*_*mean*_ the mean dose for the organ, *D*_*1%*_ the maximal dose at 1% volume for the organ. a, IMRT versus 3DCRT; b, IMRT versus RA; c, RA versus 3DCRT.

**Table 3:** Summary of the dosimetric results for PTVs, MUs and delivery time

**PTV**	**3DCRT**	**IMRT**	**RA**	**P-value**		
				**a**	**b**	**c**
V_95%_ (%)	99.73 ± 0.28	99.25 ± 1.2	99.23 ± 1.21	0.240	0.067	0.65
V_100% _(%)	80.57 ± 1.23	79.83 ± 4.01	78.56 ±3.50	0.21	0.23	0.52
V_110% _(%)	9.33 ± 8.58	3.12 ± 3.09	2.12 ±1.56	0.002	0.50	0.008
CI	0.72 ±0.03	0.83 ±0.04	0.84 ±0.05	0.000	0.633	0.000
HI	1.16 ±0.01	1.08 ±0.03	1.09 ± 0.03	0.072	0.623	0.041
MU	250.4 ± 16.20	853.2 ± 299.2	435.5 ± 134.8	0.000	0.007	0.002
Time (min)	0.92 ± 0.05	2.18 ± 1.10	0.75 ±0.13	0.000	0.000	0.332

Statistical significance (p <0.05) is reported between couples from paired *t*-test analysis. *Abbreviations:PTV* planned tumor volume, *3DCRT* 3-D conformal radiation therapy, *IMRT* intensity-modulated radiation therapy, *RA* RapidArc, *V*_*x%*_ the volume receiving ≥ x% of the prescribed dose, *CI* conformity index, *HI* homogeneity index, *MU* monitor unit. a, IMRT versus 3DCRT; b, IMRT versus RA; c, RA versus 3DCRT.

**Table 4:** Comparison of predicting parameters for RILD between smaller and larger tumors

		**3DCRT**	**IMRT**	**RA**	**P-value**		
					**a**	**b**	**c**
Dmean	D ≤ 8 cm	14.65 ± 3.12	14.32 ± 2. 90	14.30 ± 2.93	0.064	0.094	0.314
	D > 8 cm	25.31 ± 2.73	27.49 ± 2. 33	27.01 ± 2.18	0.014	0.433	0.026
V5	D ≤ 8 cm	58.30 ± 18.04	60.20 ± 17.62	66.18 ± 20.74	0.136	0.017	0.019
	D > 8 cm	81.14 ± 14.70	83.72 ± 14.07	84.82 ± 14.23	0.051	0.226	0.090
V10	D ≤ 8 cm	43.21 ± 10.09	42.50 ± 8.26	47.62 ± 11.55	0.638	0.080	0.084
	D > 8 cm	74.55 ± 20.56	78.15 ± 16.48	80.24 ± 17.61	0.359	0.074	0.189
V20	D ≤ 8 cm	26.83 ± 7.35	28.26 ± 6.92	26.08 ± 5.73	0.428	0.057	0.717
	D > 8 cm	73.20 ± 16.10	64.99 ± 17.14	61.98 ± 13.34	0.023	0.273	0.022
V30	D ≤ 8 cm	18.51 ± 5.43	13.77 ± 4.51	14.72 ± 3.67	0.34	0.157	0.024
	D > 8 cm	27.29 ± 11.32	35. 21 ± 3.57	31.17 ± 2.90	0.062	0.262	0.069

Statistical significance (p <0.05) was reported between couples from paired *t*-test analysis. *Abbreviations:3DCRT* 3-D conformal radiation therapy, *IMRT* intensity-modulated radiation therapy, *RA* RapidArc. *V*_*x*_ the volume of the OARs receiving the dose > x Gy. *D*_*mean*_ the mean dose for the organ, a, IMRT versus 3DCRT. b, IMRT versus RA; c, RA versus 3DCRT.

### Target coverage, dose homogeneity and conformity

The coverage of PTVs of the three plans were evaluated by prescribed dose (V_100%_), HI and CI. All 95% of prescribed dose could cover at least 99% of the PTV without any significant difference for three plans. The value of CI for 3DCRT (0.72 ± 0.03) was significantly lower than that of IMRT (0.83 ± 0.04) (p <0.001) or RA (0.84 ± 0.05) (p <0.001). The V_95%_ and V_100%_ values were 99.73 ± 0.28 and 80.57 ± 1.23 for 3DCRT, 99.25 ± 1.20 and 79.83 ± 4.01 for IMRT, 99.23 ± 1.21 and 78.56 ±3.50 for RA, respectively, and no significant difference was observed. HI for 3DCRT (1.16 ± 0.01) was higher than RA (1.09 ± 0.03) (p = 0.041). For the hot spot sparing, the mean V_110%_ of the PTV was significantly higher for 3DCRT (9.33 ± 8.58) than IMRT (3.31 ± 3.09) (p <0.001) or RA (2.12 ± 1.56) (p <0.001). In the typical examples in Figures 1 and 2, RA and IMRT achieved better conformity of the PTV compared with 3DCRT.

### Organs at risk

The mean dose of the normal liver for each plan was 20.57 ± 7.12 Gy for 3DCRT, 22.34 ± 7.33 Gy for IMRT, and 20.51 ± 7.12 Gy for RA. We could see a higher value of IMRT compared with 3DCRT (p = 0.045), but no difference was found between RA and 3DCRT. For the low-dose region, V_5_ was significantly highest for RA (76.34 ± 19.12) and the lowest for 3DCRT (68.90 ± 19.23), and the difference between IMRT and 3DCRT, IMRT and RA, 3DCRT and RA were respectively 0.02, 0.015 and 0.007. For V_10_, RA (64.71 ± 21.63) was higher than 3DCRT (60.37 ± 21.54, p = 0.004), IMRT (65.12 ± 21.62) was higher than RA (p = 0.031). No significant difference was observed between IMRT and 3DCRT (p = 0.274). For V_20_, RA(43.94 ± 20.10) was lower than 3DCRT (48.34 ± 21.13) (p = 0.012). For V_30_ and V_40_, 3DCRT (22.27 ± 17.30 and 27.73 ± 18.73) was higher than IMRT (22.57 ± 15.73 and 17.94 ± 10.13) (p = 0.002 and p = 0.012, respectively) or RA (21.93 ± 14.30 and 17.93 ± 10.24) (p = 0.013 and p = 0.038, respectively). In the DVHs in Figures 3 and 4, Right figure revealed similar homogeneity of the PTV for 3 plans and 3DCRT obtained highest volume of hot spot. In Figure 3, left figure showed that RA obtained the highest low-dose distribution in the normal liver compared with 3DCRT and IMRT. 3DCRT obtained the highest high-dose distribution in the normal liver compared with IMRT and RA. In Figure 4, left figure showed that the low-dose distributions for three techniques were similar. For V_20_ and V_30_, the value of 3DCRT was higher than IMRT or RA, but no statistical significance was observed (Table 4). For D_mean_ of stomach, bilateral kidneys and the maximum dose spinal cord received (D_1%_), there were no significant differences.

### Comparison of predicting parameters for RILD between smaller and larger tumors

For smaller tumors (D ≤ 8 cm), no difference was observed among three techniques for D_mean_,V_20_, and V_30_. For V_5_ and V_10_, RA (66.18 ± 20.74, 47.62 ± 11.55) was significantly higher than 3DCRT (58.30 ± 18.04 and 43.21 ± 10.09) (p = 0.019 and p = 0.017) or IMRT (60.20 ± 17.62 and 42.50 ± 8.26) (p = 0.084 and p = 0.08). For larger tumors (D > 8 cm), the D_mean_ of 3DCRT was lower (25.31 ± 2.73) than IMRT (27.49 ± 2.33) (p = 0.014) or RA (27.01 ± 2.18) (p = 0.026). For V_5_, V_10_, V_20_, and V_30_, no difference was observed among three techniques.

### Monitor units, and delivery time

The values of MUs were 250.4 ± 16.20 for 3DCRT, 853.2 ± 299.28 for IMRT and 435.5 ± 134.8 for RA with a significantly higher MUs for IMRT compared with 3DCRT (p <0.001) or RA (p = 0.007). What is more, IMRT had a much longer delivery time (2.18 ± 1.10 min) compared with 3DCRT (0.92 ± 0.05 min) (p <0.001) or RA (0.75 ± 0.13 min) (p <0.001).

## Discussion

Historically, the role of RT in HCC had been always limited for the risk of RILD. There have been efforts to identify the risk factors and the predicting parameters in the literatures that indicate increased risk of RILD after RT. In the study of Kim et al., V_30_ was demonstrated as a significant parameter in patients treated with conventional fractionated RT [19]. According to Liang et al., V_20_ was a significant parameter in patients treated with conformal radiotherapy therapy [20]. In our study, there was significantly higher V_30_ of liver for 3DCRT compared with RA (p = 0.013) or IMRT (p = 0.002). For V_20_, the values of 3DCRT was also higher than RA (p = 0.012). For V_40_ in present study, the value was higher for 3DCRT when compared with the other two plans but no significant difference was observed. Therefore, these may indicate that RA was superior to 3DCRT or IMRT at the risk of RILD in consideration of lower V_20_ and V_30_.

For the issue of higher low-dose region, a meta-analysis [21] showed that larger low-dose volume of V_5_ on total lung might contribute to radiation pneumonitis. Kim et al. [22] reported that the low-dose coverage V_5_, V_10_ to the stomach were associated with the toxicity. But the potential risk of RILD caused by low-dose irradiation is unclear. In present study, there was significant difference for V_5_ of liver among three techniques. The result was as follows: RA > IMRT >3DCRT. For V_10_, the value of RA was higher than 3DCRT (p = 0.004) while the value of IMRT was the highest (p < 0.05). These parameters should not be overlooked and the role of V_5_ and V_10_ for RILD needs to be elucidated in further studies.

There are many studies demonstrating the relationship between D_mean_ and RILD. Dawson et al. reported that a 5% and 50% probability of RILD for patients treated in their analysis were associated with the mean liver dose of 31 Gy and 43 Gy [23]. Cheng [24] et al. reported that the mean liver dose of patients with RILD was significantly higher than those without (25.04 Gy vs 19.65 Gy, p = 0.02). In consideration of the influence of PTV size to the radiation tolerance [7], we divided the patients into two groups according to median value (8 cm) of the tumor diameters. For smaller tumors (D ≤ 8 cm), no difference was observed except for higher V5 of RA compared with IMRT (p = 0.017) and 3DCRT (p = 0.019). For larger tumors (D > 8 cm), 3DCRT achieved lower D_mean_ compared with IMRT (p = 0.014) or RA (p = 0.026). But for V_5_, V_10_, V_20_ and V_30_, there were no differences. This may indicate that 3DCRT may be superior to RA or IMRT at the risk of RILD in consideration of lower D_mean_. Therefore, for larger tumors in our study, 3DCRT may be more suitable among three techniques.

Recent years, RA has gained more interest. Many studies have showed that RA can achieve superior target coverage, better conformity, shorter treatment time and less MUs compared with IMRT or 3DCRT [13,14,25]. In present study, among the three techniques, RA achieved better CI and lower V_110%_ compared with 3DCRT. The hot spots in our study were almost located on tumors, so there is not much influence of hot spot among three plans. Moreover, RA had lower V_20_ and V_30_ (p < 0.05) for liver. For V_95%_, V_100%_, mean dose of the stomach, kidneys and D_1%_ of the spinal cord, there were no significant differences for three techniques. What is more, RA achieved the lowest MUs and shortest delivery time which is in line with other reports [13,14,25]. The reduction of total treatment time may improve patients’ comfort on the couch, reduce the risk of inter-fraction movements and minimize organ displacement. But for larger tumors in our study, RA and IMRT had higher D_mean_ of liver compared with 3DCRT. What is more, the treatment of RA was much more expensive than 3DCRT.

In our study we had only 20 patients enrolled in our study which is a small sample. What is more, we did not combine each technique with respiratory gating and this might result in a proportion of the liver shifting between the high- and low-dose regions during RT.

## Conclusion

In consideration of lower V20, V30, lower MUs and shorter delivery time, RA may be superior to 3DCRT or IMRT in terms of risk of RILD for right liver lobe tumors, but for larger tumors (D > 8 cm), 3DCRT had the lowest value of D_mean_ and may be more suitable among three techniques. More clinical comparison about the predicting parameters for RILD risks are needed among different plans and this may be beneficial to HCC patients.

## Abbreviations

3DCRT: Three-dimensional conformal radiation therapy; IMRT: Intensity-modulated radiation therapy; RA: RapidArc; HCC: Hepatocellular carcinoma; OARs: Organs at risk; RILD: Radiation-induced liver disease; HBV: Hepatitis B virus; CI: Conformity Index; HI: Homogeneity index; RT: Radiotherapy; TACE: Transarterial chemoembolization; GTV: Gross tumor volume; PTV: Planning target volume; DVH: Dose-volume histograms; DR: Dose rate; MLC: Multileaf collimator; MU: Monitor units; Vx%: Volume receiving x% of the prescribed dose; Vx: Volume of the OAR receiving the dose > x Gy; Dmean: Dose mean; Vt: Volume of PTV; Vref: Volume enclosed by the prescription dose line; Vt,ref: Volume of PTV within V_ref_.

## Competing interests

The authors declare that they have no competing interests.

## Authors’ contributions

DC and RW contributed significantly to study design and concept. DC also contributed to manuscript writing and study coordinator. XM, HY and XX contributed to statistical analysis. SL, RF, XD and TL contributed significantly to the acquisition of data and optimization of treatment plans. SJ and KZ contributed to final revision of manuscript. All authors read and approved the final manuscript.
